# Unambiguous Detection of Multiple *TP53* Gene Mutations in AAN-Associated Urothelial Cancer in Belgium Using Laser Capture Microdissection

**DOI:** 10.1371/journal.pone.0106301

**Published:** 2014-09-03

**Authors:** Selda Aydin, Anne-France Dekairelle, Jérôme Ambroise, Jean-François Durant, Michel Heusterspreute, Yves Guiot, Jean-Pierre Cosyns, Jean-Luc Gala

**Affiliations:** 1 Department of Pathology, Cliniques Universitaires Saint-Luc (CUSL), Université Catholique de Louvain (UCL), Institut de Recherche Expérimentale et Clinique (IREC), Brussels, Belgium; 2 Center for Applied Molecular Technologies (CTMA), Cliniques Universitaires Saint-Luc (CUSL), Université Catholique de Louvain (UCL), Institut de Recherche Expérimentale et Clinique (IREC), Brussels, Belgium; Oklahoma University Health Sciences Center, United States of America

## Abstract

In the Balkan and Taiwan, the relationship between exposure to aristolochic acid and risk of urothelial neoplasms was inferred from the A>T genetic hallmark in *TP53* gene from malignant cells. This study aimed to characterize the *TP53* mutational spectrum in urothelial cancers consecutive to Aristolochic Acid Nephropathy in Belgium. Serial frozen tumor sections from female patients (n = 5) exposed to aristolochic acid during weight-loss regimen were alternatively used either for p53 immunostaining or laser microdissection. Tissue areas with at least 60% p53-positive nuclei were selected for microdissecting sections according to p53-positive matching areas. All areas appeared to be carcinoma in situ. After DNA extraction, mutations in the *TP53* hot spot region (exons 5–8) were identified using nested-PCR and sequencing. False-negative controls consisted in microdissecting fresh-frozen tumor tissues both from a patient with a Li-Fraumeni syndrome who carried a p53 constitutional mutation, and from *KRas* mutated adenocarcinomas. To rule out false-positive results potentially generated by microdissection and nested-PCR, a phenacetin-associated urothelial carcinoma and normal fresh ureteral tissues (n = 4) were processed with high laser power. No unexpected results being identified, molecular analysis was pursued on malignant tissues, showing at least one mutation in all (six different mutations in two) patients, with 13/16 exonic (nonsense, 2; missense, 11) and 3/16 intronic (one splice site) mutations. They were distributed as transitions (n = 7) or transversions (n = 9), with an equal prevalence of A>T and G>T (3/16 each). While current results are in line with A>T prevalence previously reported in Balkan and Taiwan studies, they also demonstrate that multiple mutations in the *TP53* hot spot region and a high frequency of G>T transversion appear as a complementary signature reflecting the toxicity of a cumulative dose of aristolochic acid ingested over a short period of time.

## Introduction

The Aristolochic Acid Nephropathy (AAN) was first reported in the early 1990's in Belgian patients having undertaken a weight-loss regimen contaminated with aristolochic acid (AA) [1], [2]. AAN is characterized by a rapidly progressive interstitial nephropathy with tubular proteinuria and glucosuria, early severe anemia, extensive hypocellular interstitial fibrosis decreasing from the outer to the inner cortical labyrinth, and a rapid development of urinary tract transitional cell carcinomas in 40–46% of the patients within 2–6 years after cessation of exposure [3]–[6]. AAN is now recognized as a devastating disease occurring worldwide with as many as 100 million people potentially at risk of AA exposure [7]. While the mechanism of AA nephrotoxicity remains to be thoroughly explored, the carcinogenic activity is currently attributed to genotoxicity of AL (aristolactam)-DNA adducts characterized by a high frequency of A>T transversion in the *TP53* tumour suppressor gene of AA-associated tumors. This has been well documented in animal experiments [8], [9] and, although not consistently, in patients showing clinical and histopathological similarities with the original Belgian AAN cases [10]–[14]. Whereas AL-DNA adducts demonstrating AA exposure have been well documented in kidney tissues from the Belgian cohort [6], [15], [16], the presence of A>T transversion witnessing the causal relationship between exposure and malignancy had yet to be investigated. The aim of the present work was therefore to characterize the *TP53* mutational spectrum in frozen samples of malignant urothelial tissues from Belgian AAN patients using thoroughly validated genotyping methods.

## Patients and Methods

### Ethics Statement

In 2001, a study on the genetic polymorphism of enzymes implicated in the metabolism of aristolochic acid among Belgian AAN patients was approved by Prof JM Maloteaux, Chairman of the Faculty of Medicine and Health Sciences Research Ethics Committee of the Université catholique de Louvain (Belgium). Following Ethics Committee approval, a written informed consent was provided by each patient from the Belgian AAN series. These study data remained unpublished.

In 2009, an amendment to the pilot study defined specifically the current *TP53* research which was carried out on tissue samples from 5 AAN patients reported in this study, and was accepted as such by the Research Ethics Committee of the Université catholique de Louvain (Belgium) as a continuation of the study started in 2001. Tissue samples from patients 1–5 which were all part of the original study (2001) were anonymized prior to analysis.

Blood samples were part of a previous study (UCL Ethics Committee approval reference no. 2003/05/03/08) [17]. Adenocarcinoma and surgical ureteric samples were collected from AA-unrelated patients as part of their medical treatment and were anonymized prior to analysis. After written information, adenocarcinoma tissues were stored in UCL Biological library (http://www.centreducancer.be/fr/show/index/section/8/page/34). Non AA-related ureteric samples taken from nephroureterectomies removed at the time of renal transplantation were used as controls in this study with a codification after pathological analysis in accordance with the Belgian laws on tissue banking (2008).

The snap-frozen granulosa-cell tumour specimen from the Li-Fraumeni patient was obtained from the UCL biological library (same as above). After written consent from the parents, this sample was anonymized prior to use.

Previous reported data include AL-DNA adducts identified in kidney tissue from patients 1, 3, 4 [15], [16], [18] and *TP53* gene sequencing performed in a bladder TCC from patient 1 [10].

### AAN patients

Five AAN patients referred to Cliniques Universitaires Saint-Luc (Brussels, Belgium) were studied (Table 1). All were women with a mean age of 42.8 years (range: 27–53) at presentation. One patient was a smoker. None had a history of analgesic abuse or typical features of analgesic nephropathy. They all underwent bilateral nephroureterectomy either at the time of renal transplantation or subsequently for upper urinary tract malignancies. Besides, one patient also underwent transurethral resections of recurrent transitional cell carcinoma (TCC) of the bladder and, eventually, cystectomy. The diagnosis of AAN was based on the following criteria: AA intake through a weight-loss regimen phytochemically demonstrated as contaminated (patients 1, 2, 3 and 5), a typical renal histology of interstitial fibrosis (all five patients), identification of AL-DNA adducts in kidney tissue (patients 1, 3 and 4) and development of upper urinary tract malignancy (all five patients) [15], [16], [18]. In patient 4, the intake of AA could however never be formally proven as she claimed to have attended the weight-loss clinic before the regimen contaminated with AA (so-called “formula 2”) was introduced [1]. Nevertheless, all five patients fulfil the recently proposed criteria for a definite diagnosis of AAN [19] in the current case series. Albeit lacking the *TP53* mutation status, it is worth noting that patient 1 is n°3 in references 4, 5, 15 and 16, patient 2 is n°7 in reference 5, patient 3 is n°4 in references 15 and 16, n°5 in reference 5, and patient 4 is n°2 in reference 20 and number n°8 in reference 18 whereas patient 5 has not yet been reported.

**Table 1:** Clinical, pathological and *TP53* gene mutational data of areas with extensive p53 IHC overexpression in TCC of Belgian AAN patients.

*AAN Patients (Age/Sex)*	*Duration of formula 2 regimen (month)*	*Smoking status (pack-years)*	*Months from end of AA exposure and Surgery*	*RAL in renal tissue (mean ± SD/10exp7 nucleotides)^a^*	*p53 positive areas analysed for TP53 mutations*							
					*Localisation of tissue*	*Multifocal TCC*	*IHC*	*Intron*	*Exon*	*Genomic position*	*Mutated codon*	*Effect of the mutation*
1 (27/F)	20	5	15 (Left Nux)	4.1±2.7		-	ND	ND	ND	ND	ND	ND
			27 (Right Nux)	3.0±1.8	Pelvis	CiS	ND	ND	ND	ND	ND	ND
			111 (Cx)*	ND	Bladder	CiS and papillary	+	-	7	g. A13325C	T230P	Missense
										g.G13380A	R248Q	Missense
2 (41/F)	15	0	50 (Left Nux)	ND	Pelvis	CiS	+	-	5	g.A12478T	K164X	Nonsense
			53 (Right Nux)	ND	Pelvis	CiS	+	-	-	-	-	-
3 (42/F)	21	0	18 (Right Nux)	2.5±2.1	Ureter	CiS	ND	ND	ND	ND	ND	ND
			64 (Left Nux)	ND	Pelvis	CiS	+	7	-	g.G13757C		Intronic
4 (53/F)	NA	0	NA (Bilateral Nx)	2.9±2.0 in right cortex		-	ND	ND	ND	ND	ND	ND
			NA (Right subtotal Ux)	ND	Upper ureter	CiS	ND	ND	ND	ND	ND	ND
			NA (Left Ux and remnant right° Ux)	ND	Left upper ureter	CiS	+	-	5	g.C12401T	A138V	Missense
							+	-	6	g.T12722C	V218A	Missense
					Left mid ureter	CiS	+	-	5	g.G12461T	R158L	Missense
							+	6	-	g.G12759A		Intronic
							+	-	7	g.G13323T	C229F	Missense
					Left lower ureter	CiS	+	-	8	g.C13824T	R282W	Missense
5 (51/F)	20	0	96 (Right and left° Nux)	ND	Right pelvis	CiS	+	-	6	g.A12728G	Y220C	Missense
									8	g.G13791C	E271Q	Missense
										g.A13837T	E286V	Missense
					Right mid ureter	CiS	+	5	-	g.A12627T		Donor splice site
								-	6	g.A12683G	Y205C	Missense
								-	8	g.G13836T	E286X	Nonsense

Abbreviations: RAL: Relative adduct labelling, Nx: Nephrectomy, Nux: Nephroureterectomy, Ux: Ureterectomy, Cx:Cystectomy, ND: not done, NA: not available.

a Mean of at least three determinations in reported separate experiments [15], [16], [18].

* Mutations previously reported [10] and confirmed by the FASAY method [25] in the Center for Applied Molecular Technology of the Université Catholique de Louvain.

° No CiS found.

### Surgical specimens

Tissues from five pelvi-ureterectomies and a single cystectomy were selected on the basis of the availability of frozen material. Carcinoma *in situ* (CiS) and papillary TCC were diagnosed accordingly to the 2004 WHO classification of urothelial tumours [21], [22]. Unilateral multifocal CiS developed in the right upper urinary tract (pelvis, upper, mid and lower ureter) in two patients (patient 1 and 5). While ureteral CiS invaded focally the lamina propria in patient 1, she also developed bladder papillary TCC. Bilateral multifocal CiS developed in the upper urinary tract of the 3 remaining patients. Delay between end of exposure and surgery averaged 44.75 months (range 15–96) in patients 1, 2, 3, 5 and was unavailable in patient 4. In the former four patients, unilateral and bilateral TCC were diagnosed an average of 61 months (range: 27–96) and 41 months (range: 18–64) after end of known exposure, respectively.

### p53 immunohistochemistry (IHC)

CiS was identified by light microscopy on hematoxylin eosin stained sections from frozen pelvi-ureteric samples. Out of serial frozen sections (7 µm-thick), sections n°1, n°3 and n°5 were used for p53 immunohistochemistry (IHC) and kept overnight at 37°C whereas sections n°2, n°4 and n°6 were used for laser microdissection. The latter three sections were placed on biochemically inert Polyethylene naphtalate (PEN) membrane covered slides and kept at -20°C until use. For p53 IHC, the sections kept at 37°C were subsequently fixed in formaldehyde 4% for 3 hours. Endogenous peroxydase was blocked with 0.3 % hydrogen peroxide in deionized water for 30 min. The slides were incubated at 97°C for 75 min and rinsed in a solution containing deionized water and triton 0.05%. The sections were then covered for 30 min with 10% normal goat serum (NGS) containing 1% bovine serum albumin (BSA), diluted in tris-triton. They were incubated overnight at room temperature with the anti-p53 mouse monoclonal antibody DO-7 (Biocarta, Europe Gmbh) at a dilution of 1∶1000. After washing with tris-triton 0.05%, the slides were incubated at room temperature for 75 min with a ready-for use anti-mouse EnVision-Peroxidase system (Dako, Glostrup, Denmark) according to the manufacturer's protocol and counterstained with hematoxylin. A normal goat serum was used as negative control.

Overexpression of p53 was defined as nuclear staining irrespective of IHC intensity. Extensive p53 staining by IHC has previously been recommended to enhance the mutation detection rate of *TP53* [23]. Accordingly, only tissue areas containing more than 60% of positive nuclei were selected for microdissection. Exactly matching areas from yet unprocessed serial sections (n°2, n°4 and n°6) underwent microdissection as detailed hereafter.

### Laser capture microdissection

**Figure 1:**
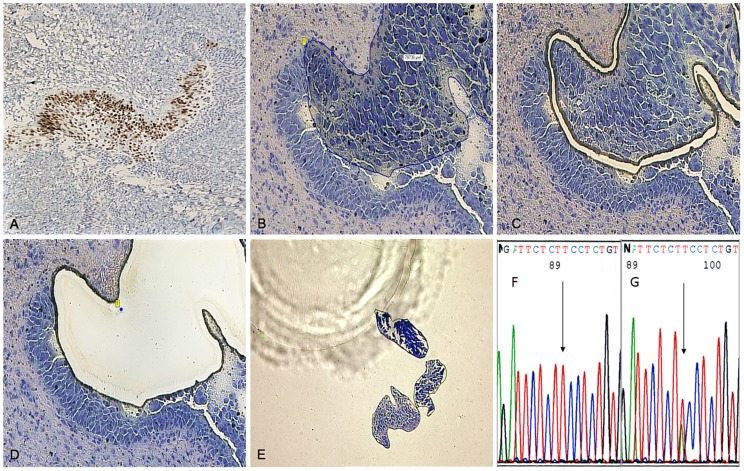
Microdissection procedure of p53 overexpressing fresh frozen malignant area. A–G TCC area containing more than 60% p53 stained nuclei by IHC counterstained with hematoxylin from frozen section of ureter (A). For laser capture microdissection, a toluidine blue stained area in a representative frozen section was matched (B), cut (C) and catapulted (D) in the cap of a microtube (E). 3′-5′dideoxy sequencing electropherogram of the segment of p53 showing the wild type sequence (F, arrow) and A>T transversion (G, arrow) leading to missense mutation E286V in exon 8.

After toluidine blue staining, each unfixed frozen tissue area which exactly matched the area of interest defined according to p53 IHC slides was isolated using a PALM microlaser system (Bernried, Germany) equipped with a pulsed UV nitrogen laser (wavelength: 337 nm; Pulse energy >270 µJ, pulse duration 3nsec, pulse frequency 1–30/sec) and the PALM Robot Software Version 2.2. The system was coupled to an Axiovert 200 microscope and a Plan Neofluar 20× (Zeiss, Oberkochen, Germany). Microdissected foci were catapulted into a microtube cap and frozen at −20°C until DNA extraction (Figure 1).

### DNA extraction

In each cap, 10 µl of a solution containing EDTA 0.001 M (pH 8), Tris HCl 0.02 M (pH 8), 0.5% Tween 20, proteinase K (2 mg/ml) and ultrapure water was included. After mixing and centrifugation, each reaction tube was overlaid with one drop of mineral oil to prevent evaporation and incubated overnight at 55°C with continuous agitation (600 rpm). Ultrapure water (5 µl) was then added to the reaction tube to increase the final volume of the solution. After centrifugation, the solution was heated at 99°C for 10 min to inactivate the proteinase K. Finally the extract was transferred to another microtube.

### Nested-PCR

Exons 5 to 8, corresponding to the p53 DNA binding domain, a so-called “hot spot” for *TP53* gene mutations [24], were amplified by nested-PCR. For the first PCR round, 3 µl of DNA extracted from each microdissected sample was added to a final 50 µL PCR mix including 2.25 mM MgCl_2_, 0.2 U/μL Taq Gold polymerase (Ampli Taq Gold, Applied Biosystems, Roche), 0.2 mM dNTP and 0.2 pm/μL primers (Eurogentec, Liège, Belgium) (Table 2). An initial incubation at 95°C for 4 min was followed by 25 cycles (95°C for 30 sec, 60°C except for exon 7 where annealing temperature was 55°C for 30 sec and 72°C for 1 min) and a final elongation for 7 min at 72°C. The second PCR round was carried out with 2.5 µl of the first PCR product using the same conditions as for the first round, except that 1.5 mM of MgCl_2_ and 0.4 U/μL of Taq Gold polymerase were used for exon 6. Ultrapure water was used as negative control whereas DNA extracted from unrelated blood samples was used as control for *TP53* amplification.

**Table 2:** Primers used in the nested polymerase chain reaction.

Exons	Primers	Sequence (5′-3′)	Exon Position	Target size (bp)	Concentration (nm)
					
5	E-S	TGTTCACTTGTGCCCTGACTT	12334 -	21	60.7
	I-S	TTCAACTCTGTCTCCTTCCTCTTC	12357-…	24	128.55
	E-AS	AGAGCAATCAGTGAGGAATCAG	12604-…	22	60.87
	I-AS	AGCCCTGTCGTCTCTCCA	12563-…	18	113.69
6	E-S	TTGCCCAGGGTCCCCAG	12599-…	17	78.38
	I-S	GGCCTCTGATTCCTCACTGA	12618-…	20	40.04
	E-AS	GAGGGCCACTGACAACCA	12784-…	18	77.91
	I-AS	TTAACCCCTCCTCCCAGAGA	12761-…	20	51.3
7	E-S	GCCACAGGTCTCCCCAA	13270-…	17	72.1
	I-S	GCGCACTGGCCTCATCT	13288-…	17	104.67
	E-AS	GGTCAGCGGCAAGCAGA	13468-…	17	48.13
	I-AS	TGCAGGGTGGCAAGTGG	13428-…	17	102.96
8	E-S	GGTTGGGAGTAGATGGAGCCT	13690-…	21	29.54
	I-S	TTTCCTTACTGCCTCTTGCTTCT	13743-…	23	106.47
	E-AS	CATTTTGAGTGTTAGACTGGAAACTTT	14097-…	27	53.77
	I-AS	TGAGGCATAACTGCACCCTT	13935-…	20	74.94

### Sequence analysis

Amplified products were purified using the MSB Spin PCRapace kit (STRATEC Molecular GmbH, Berlin, Germany) according to the manufacturer's instructions. Sequence analysis was carried out on an automated ABI 377 A apparatus (Applied Biosystems, Foster City, CA), using the Taq Dye Deoxy Terminator Cycle Sequencing kit from the same manufacturer and according to its instructions. The *TP53* reference sequence from Genbank was NC_000017.10.

### Assessment of potential *TP53* -artifactual alterations in tissue samples

(a) To assess the ability of the current microdissection procedure to identify DNA mutations, four colo-rectal adenocarcinomas previously investigated for *KRas* mutation by routine clinical testing on FFPE (Formalin-fixed paraffin-embedded) tissue specimens were selected. Two of them carried a mutation and two were wild-type. These four samples were chosen because a snap-frozen counterpart was kept stored at −70°C in the Cancer Human Bio-bank of the Université catholique de Louvain (https://www.uclouvain.be/416846.html). Laser microdissection and DNA extraction of snap-frozen adenocarcinoma specimens were carried out using the procedure described above and the results of *KRas* testing were compared. Additionally, a snap frozen granulosa-cell tumour specimen from a 15 year-old young girl with a Li-Fraumeni syndrome was analysed using the same microdissection procedure. While the constitutional mutation had already been identified by FASAY (functional analysis of separated alleles in yeast) of the *TP53* mRNA extracted from peripheral mononuclear cells of the patient [25], the tumour specimen was studied to further confirm the ability of the current microdissection procedure to identify correctly this *TP53* mutation in frozen sections of the tumour.

(b) Whether snap-frozen tissue sections could be affected by a laser-induced effect is not known and this could even be worsened by additional interfering factors such as a deleterious effect of the fixation, staining and/or nested-PCR procedures. To exclude such technological bias, surgical ureter specimens were collected after nephroureterectomies (n = 4) taken from kidney transplanted patients for terminal uremia due to polycystic kidney disease (n = 2), chronic idiopathic interstitial nephritis definitely unrelated to AA ingestion (n = 1), and diabetic glomerulosclerosis (n = 1). These specimens were immediately embedded in Tissue-Tek and snap-frozen, and urothelial areas were processed concomitantly in three different ways. Microdissection was carried out under two different intensities (low mean UV-energy: 68; and high mean UV-energy: 69.4) and subsequently processed as reported here for the AAN-patient samples. By comparison, additional serial sections from the same ureter sample underwent a simple slide scratching as carried out in *KRas* routine mutation testing on FFPE tissue specimens. DNA extraction, amplification and *TP53* sequencing were carried out according to the method described in this article.

(c) As additional control of AA-unrelated *TP53* artifactual alterations potentially induced by the current analysis, a phenacetin-induced urothelial carcinoma which is reportedly characterized by the absence of A > T transversions in the *TP53* gene [26] was removed from the lower ureter of a 64 year-old female, embedded in Tissue-Tek, snap-frozen in liquid nitrogen-cooled 2-methylbutane (isopentane) and stored at −70°C. IHC, microdissection, DNA extraction and *TP53* sequencing were performed according to the methods described above.

### Assessment of limit of detection for Sanger sequencing

For the limit of detection study, DNA was extracted from two Head and Neck Squamous Cell Carcinoma cell lines (SC173 and SC263, kindly provided by A.C. Begg, Netherlands Cancer Institute, The Netherlands) carrying different *TP53* mutations and from a wild-type *TP53* HNSCC cell line (HN30, kindly provided by M. Flinterman, Department of Oral Medicine and Pathology, King's College London, The Rain Institute, UK). Characterization of the *TP53* status was previously performed in our laboratory using FASAY analysis [25]. SC173 carries a missense mutation in exon 5: R175H (CGC>CAC). SC263 presents a compound heterozygous alteration involving a nonsense mutation R306X in exon 8 (CGA>TGA) and a 32-bp deletion in exon 7 (del 704–735) (CTMA laboratory data). DNA from both *TP53* mutated cells was serially diluted and spiked in the wild-type cell DNA to have 50 to 1.25% mutant to wild-type DNA ratios. Amplification and sequencing analysis were carried out as described above.

### Comparative statistical analysis of number and type of mutations in current and previous data

A Poisson regression model was built to compare the prevalence and type (A>T and G>T) of *TP53* mutations in p53 binding site between current (n = 5) and previous [BEN (n = 11 and n = 97) and Taiwanese (n = 151)] clinical series [11], [12], [14], and to compare the results of this analysis with those from patients with urothelial malignancies (bladder, ureter, upper urinary tract and renal pelvis) (n = 1111) as reported in the *TP53* IARC database [27]. It is worth noting that data associated with AA exposure were discarded from *TP53* IARC database results.

## Results

### Assessment of potential artifactual *TP53* alterations in tissue samples

Using the microdissection procedure enabled a correct identification of known DNA mutations characterized by previous routine testing. Regarding the identification of *KRas* mutations on snap-frozen adenocarcinomas, results were strictly identical to those previously obtained on FFPE samples (i.e., identification of G12S and G12D mutations in the *KRas* mutated tissues and wild-type status in the other two tumours). Regarding identification of a constitutional g.13380 G>A, p.R248Q mutation identified by FASAY assay in peripheral blood cells of a patient with Li-Fraumeni syndrome, this mutation was also found in malignant cells from the granulosa cell tumor using DNA extraction, microdissection, nested-PCR and sequencing procedures as used in the present study.

Conversely, no artifactual *TP53* alterations were found in the various controls carried out to test the occurrence of *TP53* DNA-induced damage related to DNA extraction, microdissection, nested-PCR and sequencing procedures. No mutations were found in three out of four ureter samples used to test a *TP53* iatrogenic damage. The only exception was the ureter sample from a type 2 diabetic patient with a G>A transition (g.12455, p.R156H) in exon 5 of the *TP53* gene after microdissection under high laser power. No mutations were found in the p53 DNA binding domain (exons 5 to 8) when analyzing DNA extracted from a phenacetin-induced urothelial carcinoma.

### Assessment of limit of detection for Sanger sequencing

The limit of detection for Sanger sequencing was identified at 6.25% of mutant to wild-type DNA ratio with both *TP53* mutated cell lines, irrespective of the mutation or deletion assessed. At this DNA ratio, the heterozygous A/G peak with serial DNA dilutions from SC173/HN30 mixed DNAs was still visible as also was the heterozygous T and C peak combined with a 32-bp deletion in exon 7 with serial DNA dilutions from SC263/HN30 mixed DNAs.

### Morphology

All p53 positive areas corresponded to papillary TCC or to CiS containing anaplastic and dysplastic cells (Table 1). Intra-urothelial and lamina propria inflammation, hyperplasia and reactive atypia were absent. In patient 2, neoplastic and dysplastic cells were clinging to the basement membrane (clinging CiS).

### TP53 gene mutations

Including two previously reported results [10], a total of 16 totally different exonic (n = 13) or intronic (n = 3) mutations of the *TP53* gene were found in malignant urothelial tissues from the Belgian AAN cohort (Table 1). In patient 1, two mutations were found in the same exon (exon 7) whereas a single mutation was found in patient 2 (exon 5) and 3 (intron 7). Patients 4 and 5 harbored 5 different exonic and 1 intronic mutations. Among the 13 exonic mutations, two were nonsense (patients 2 and 5) and 11 missense mutations (patients 1, 4 and 5). They were located in exon 5 (3 mutations), exon 6 (3 mutations), exon 7 (3 mutations) or exon 8 (4 mutations). One of the three intronic mutations (g.12627 A>T, patient 5) was located at the AG acceptor splice site. Globally, mutations affected A∶T pairs (7/16) and G∶C pairs (9/16). There was a nearly equal number of transitions (7/16) and transversions (9/16). Transitions (7/16) occurred indeed with a similar frequency at A∶T pairs (3/16) and G∶C pairs (4/16). A>G, G>A and C>T (2/16) occurred with a similar frequency as T> C (1/16). Similarly, transversions (9/16) were found at A∶T pairs (4/16) and G∶C pairs (5/16) distributed as follows: A>T (3/16), G>T (3/16), G>C (2/16) and A>C (1/16).

### Comparative statistical analysis of number and type of mutations in current and previous data

The Poisson regression model showed a highly significant (p<0.001) relative increase of the *TP53* mutation prevalence in p53 hotspot codons from the current clinical series, compared with IARC database results (Table 3). In contrast, a significant relative decrease was found both in the BEN (n = 97) [12] and the Taiwanese (n = 151) [14] clinical series compared with IARC and current clinical data (Table 3). A non-significant (p>0.05) relative decrease/increase was found in BEN (n = 11) [11] compared with IARC and current clinical data (Table 3).

**Table 3:** Relative increased/decreased prevalence of mutation.

Reference database	Compared database			
	BEN (n = 11) [11]	BEN (n = 97) [12]	Taiwan (n = 151) [14]	BELGIAN (n = 5)
**IARC (n = 1111)**	1.54 [0.98–2.42], p = 0.062	0.46 [0.35–0.61], p<0.001	0.50 [0.40–0.62], p<0.001	2.85 [1.74–4.67], p<0.001
**BELGIAN (n = 5)**	0.54 [0.28–1.05], p = 0.069	0.16 [0.09–0.28], p<0.001	0.17 [ 0.10–0.30], p<0.001	

Regarding the number of A>T transversion per patient, a significant relative increase was found in the current, both BEN [11], [12], and the Taiwanese [14] clinical series, compared with IARC database results (Table 4). Non-significant (p>0.05) results were obtained when previous AAN and current clinical series were compared (Table 4).

**Table 4:** Relative increased/decreased prevalence of A>T mutation.

Reference database	Compared database			
	BEN (n = 11) [11]	BEN (n = 97) [12]	Taiwan (n = 151) [14]	BELGIAN (n = 5)
**IARC (n = 1111)**	40.40 [21.74–75.09], p<0.001	10.80 [6.71–17.38], p<0.001	9.67 [6.23–15.01], p<0.001	19.05 [5.86–61.93], p<0.001
**BELGIAN (n = 5)**	2.12 [0.61–7.38], p = 0.23	0.57 [0.17–1.85], p = 0.34	0.51 [0.16–1.63], p = 0.26	

Compared with the IARC database results, a significant 5.85 increase of G>T prevalence was found in the current clinical series (Table 5). No G>T transversion was found in both BEN series. Regarding the Taiwanese series, there was a non-significant relative decrease in G>T prevalence compared with IARC data whereas such decrease was highly significant when compared with current results (Table 5).

**Table 5:** Relative increased/decreased prevalence of G>T mutation.

Reference database	Compared database			
	BEN (n = 11) [11]	BEN (n = 97) [12]	Taiwan (n = 151) [14]	BELGIAN (n = 5)
**IARC (n = 1111)**	0 *	0 *	0.65 [0.34–1.23], p = 0.18	5.85 [1.86–18.40], p = 0.002
**BELGIAN (n = 5)**	0 *	0 *	0.11 [0.03–0.40], p<0.001	

* No record of GT mutation in BEN series [11], [12].

## Discussion

This is the first report of the mutational spectrum of *TP53* tumor suppressor gene in a series of Belgian patients (n = 5) with documented AAN and subsequent development of TCC in the upper urinary tract together with bladder involvement in one of them. Four of them had followed AA-contaminated weight-loss diet and one denied exposure to AA while presenting a typical renal histology of interstitial fibrosis [20] with AL-DNA adducts [18].

Using exclusively frozen samples was an absolute prerequisite to allow adequate comparison with reported data. Except for five out of 11 patients for whom formalin-fixed paraffin embedded tissues were used [11], all major *TP53* genetic investigations in cases presenting with AA-induced genotoxicity and urothelial malignancies were indeed led using fresh frozen tissues [11], [12], [14]. Conversely, the aim was to avoid the well-known deleterious effect of formalin fixation in terms of DNA quality and characterization of tissue mutational status [28]. To prove that *TP53* mutations did not result from technological artifacts when using frozen section, a special attention was paid to thorough validation procedures. The current *TP53* testing included successive steps (i.e., laser capture microdissection of tumor cells after toluidine blue staining, DNA extraction, nested-PCR amplification and sequencing). This method did not produce false-negative results. It enabled indeed a correct identification of known *KRas* oncogenic mutations in adenocarcinomas. It also enabled a correct identification of a previously characterized *TP53* constitutional mutation associated with a Li-Fraumeni syndrome. This mutation was found in tumor cells whereas the previous characterization was carried out by a functional assay on peripheral mononuclear cells.

Likewise, the current procedure did not create false-positive results when analyzing normal ureter specimens collected after nephroureterectomy in patients with AA-unrelated diseases or a phenacetin-induced carcinoma, and taking current conventional genotyping as gold standard. This also applied when performing laser capture microdissection with high intensity. The sole mutation found was a single G>A transition (g.12455, p.R156H) in the tissue specimen from a type 2 diabetic patient without any history of neoplasia. Of interest, there is no significant difference in the prevalence of G>A transition between the general population and type 2 diabetic patients [29]. As assessed by serial dilutions of *TP53* mutated DNA, the detection limit of the current genotyping procedure was 6.25% of mutant to wild-type ratio.

Of interest, all patients from the current series were women and each of them presented at least one *TP53* mutation in their malignant urothelial tissues whereas a more balanced male/female ratio was found (53%/47%) in previous studies [11], [14]. In previous studies, the reported prevalence of patients with *TP53* hotspot mutation varied from 100% (11/11 BEN patients) [11] to 37% (36/97 BEN tumors) [12] or 47% (71/151) Taiwanese AA-patients [14]. Considering that only a single patient was reported with a quintuple mutation in the IARC database, the current observation that 40% (2/5) of the patients harbored multiple *TP53* mutations, each with six distinct mutations (five exonic and one intronic mutations, among which one splice site mutation in patient 5) was a striking and very unusual finding. It is also interesting to note that this quintuple mutation was also found in the BEN series [12] which is one of the hitherto largest reported human series of AA related *TP53* mutations. Compared to data from non AA-related urothelial cancers in the IARC database (n = 1111) and from the Taiwanese series (n = 151), the current series showed the highest relative increased prevalence of mutation and G>T transversion in the *TP53* hotspot region. Irrespective of the current or previous AA-series, the prevalence of A>T transversion was significantly and consistently higher than in the IARC database [27]. Our finding confirms that the number and profile of *TP53* mutations per patient is highly unusual, probably reflecting a sudden highly toxic exposure. Whether these multiple mutations occurred in different malignant clones was not assessed. However, the number of distinct tumor areas associated with this complex mutational spectrum certainly reflects wide within-tumor heterogeneity with poly- or multiclonal malignant urothelial cells.

Looking into details of the type of hotspot mutation in this study, point mutations at A∶T pairs were as frequent as those at G∶C pairs (7/16 versus 9/16, respectively). They were dominated by transversions with A>T and G>T accounting each for 18.7% (3/16) of all *TP53* mutations. Interestingly, A>T transversion in human AA-related urothelial tumour was first reported in a UK patient [30] but A>T transversion was then frequently found in the p53 hotspot region from urothelial carcinoma associated with the Balkan endemic nephropathy (BEN) [11], [12], as well as in Taiwan [14]. In all these studies, the population was exposed to AA for many years, with figures as high as 66% (33/50) to 72% (13/18) and 55% (46/84) of all *TP53* mutations, respectively. In the current series, one tumour with multiple *TP53* mutations harbored two A>T transversions, a feature also commonly reported in the BEN and Taiwanese series [11], [12], [14]. Animal studies further confirmed the high frequency of A>T transversions in AA-induced tumours, for instance in the forestomach epidermoid neoplastic cells from AA-exposed rats at codon 61 of the Ha-ras proto-oncogene [8], as well as in human p53 knock-in (Hupki) mouse embryonic fibroblasts exposed to AAI (Aristolochic Acid I) [31], [32]. Altogether, these findings strengthen the hypothesis that A>T transversion is both a specific alteration occurring after experimental exposure to AA in animals, and the hallmark of AA-exposure in humans. Accordingly, this genetic alteration appears to be one of the main mechanisms of carcinogenesis initiated by DNA polymerase-mediated incorporation of deoxyadenosine monophosphate (dAMP) opposite adenine adducts [33].

In addition, a high frequency of G>T transversions was also observed in the current series. The prevalence of G>T transversion is usually associated with tobacco exposure as evidenced in the Hupki mouse embryonic fibroblasts exposed to the prominent tobacco-derived carcinogen benzo[a]pyrene [34], as well as in smoking-related human lung cancers [35]. However, G>T transversions are rarely identified (4%) in animal models of AA intoxication [36], and are only occasional in Hupki mouse embryonic fibroblasts exposed to AA [31], [32]. In line with these observations, the prevalence of G>T transversion in AA-associated human urothelial cancers in Taiwan is low (12%) and found in none of both BEN series [11], [12], [14]. In the current series comprising only female patients, the statistically significant higher frequency of non-smoking associated G>T transversions compared with IARC database and other AAN clinical series [11], [12], [14], and the unusual occurrence of two G>T transversions in the same sample were therefore extremely unusual findings. They may well be consistent with the reported covalent binding of AA to d-guanosine in rodents [9] as well as in AAN patients [6], [11], [13], [16].

Consequently, this unusually high G>T frequency in the *TP53* hot spot region has to be considered at the light of major differences between previous studies [11], [12], [14] and the current work. Firstly, a limited number of patients (n = 5) was assessed in this study compared with previous studies which included 11 to ∼100 patients exposed to AA. Secondly, male and female patients were almost equally distributed in BEN and Taiwanese cohorts whereas the current cohort consisted only of women. Furthermore, there were substantial differences between both former and current cohorts in terms of mean age (66 versus 43 years old, respectively), and exposure duration and intensity. For the latter two features, no precise data could unfortunately be retrieved from the Taiwanese series as patient exposure resulted from intermittent ingestion of herbal remedies containing or likely to contain AA [14], [37]. In contrast, repeated intake of contaminated bread over time was estimated to expose BEN patients to a toxic dietary exposure which is equivalent to that documented for Belgian patients with AAN [11], [38]. While cumulative doses are a known significant risk factor for developing urothelial carcinoma [6], repeated AA exposure over a short period of time (mean 19 months) in the Belgian series may already have led to the cumulative toxic dose which, comparatively, was only achieved after several decades in BEN patients. Considering the higher daily intake of AA in Belgian compared to the BEN series and the susceptibility of hotspot codons to carcinogen-induced alterations, differences in the mutation profile would not be surprising. One of the major phenotypic difference observed in the Belgian cohort was the substantially quicker development of nephrotoxicity (within few months) and of cancer (within 2–6 years) after cessation of AA exposure [10]. Additional differences between studies laid in the current methodology for *TP53* genotyping. This was indeed carried out only on fresh tissues using tissue microdissection followed by nested-PCR and conventional sequencing, which differs significantly from the AmpliChip p53 microarray on fresh [11], [12], [14] and on few formalin-fixed tissues [11] as previously reported. In that respect, it is interesting to note that the spectrum of *TP53* genetic alterations in Taiwan and BEN patients whom tumors were assessed using identical *TP53* genotyping, was notably similar [11], [12], [14].

In conclusion, our documentation of expected A > T transversions attributable to AA exposure in the *TP53* gene of the Belgian AAN associated TCC is the first demonstration of a clear causal relationship between AA exposure and the development of urothelial malignancy in this cohort. Interestingly, and although assessed on small series of female patients, there are two striking highly significant observations characterizing the current series: the addition of poly- or multiclonal *TP53* alterations to the otherwise well-known AA mutational fingerprint and the unusually high prevalence of G>T transversion in the p53 binding site, two new features appearing as a complementary signature possibly reflecting the toxicity of a cumulative dose of AA over a short period of time.
